# Discrimination of the Red Jujube Varieties Using a Portable NIR Spectrometer and Fuzzy Improved Linear Discriminant Analysis

**DOI:** 10.3390/foods11050763

**Published:** 2022-03-07

**Authors:** Zuxuan Qi, Xiaohong Wu, Yangjian Yang, Bin Wu, Haijun Fu

**Affiliations:** 1 School of Electrical and Information Engineering, Jiangsu University, Zhenjiang 212013, China; 2221907084@stmail.ujs.edu.cn (Z.Q.); wxh419@ujs.edu.cn (X.W.); fuhaijun21@ujs.edu.cn (H.F.); 2 High-Tech Key Laboratory of Agricultural Equipment and Intelligence of Jiangsu Province, Jiangsu University, Zhenjiang 212013, China; 3 Research Institute of Zhejiang University-Taizhou, Taizhou 317700, China; 4 Department of Information Engineering, Chuzhou Polytechnic, Chuzhou 239000, China; wubin2003@163.com

**Keywords:** red jujube, near-infrared spectroscopy, feature extraction, fuzzy set theory, classification

## Abstract

In order to quickly, nondestructively, and effectively distinguish red jujube varieties, based on the combination of fuzzy theory and improved LDA (iLDA), fuzzy improved linear discriminant analysis (FiLDA) algorithm was proposed to classify near-infrared reflectance (NIR) spectra of red jujube samples. FiLDA shows performs better than iLDA in dealing with NIR spectra containing noise. Firstly, the portable NIR spectrometer was employed to gather the NIR spectra of five kinds of red jujube, and the initial NIR spectra were pretreated by standard normal variate transformation (SNV), multiplicative scatter correction (MSC), Savitzky-Golay smoothing (S-G smoothing), mean centering (MC) and Savitzky-Golay filter (S-G filter). Secondly, the high-dimensional spectra were processed for dimension reduction by principal component analysis (PCA). Then, linear discriminant analysis (LDA), iLDA and FiLDA were applied to extract features from the NIR spectra, respectively. Finally, K nearest neighbor (KNN) served as a classifier for the classification of red jujube samples. The highest classification accuracy of this identification system for red jujube, by using FiLDA and KNN, was 94.4%. These results indicated that FiLDA combined with NIR spectroscopy was an available method for identifying the red jujube varieties and this method has wide application prospects.

## 1. Introduction

Red jujube is a kind of agricultural product with a long history. It has caught the fascination of people all over the world and is widely planted in China. Red jujube is rich in a variety of nutrients that are beneficial to the human body, including sugars, fats, organic acids, amino acids, vitamins, flavonoids, and a variety of trace elements, which can prevent cancer, cardiovascular and cerebrovascular diseases [1]. For different origins of red jujube, their taste and nutritional value have obvious differences [2]. However, the current testing methods for red jujube varieties at the markets are too complicated and are unsuitable for large-scale application. Furthermore, these methods are not friendly to consumers, so it is very necessary to build a fast, concise, cheap, and reliable method that can recognize the red jujube varieties.

Some traditional identification methods of red jujube varieties have been extensively employed. Professional jujube discriminators can identify the type of red jujube by its shape, colour, and clarity. However, many professionals are vulnerable to the environment and physical state. Furthermore, it also takes plenty of time and money to train a professional red jujube appraiser. In recent years, domestic and foreign researchers actively established some methods for identifying red jujube varieties. For example, Wang et al. explored the electrical characteristics of red jujube fruits for variety identification in 2014 [3].

At present, NIR spectroscopy technology has been quite mature with the emergence of several new types of spectral instruments, and there it has many advantages: fast, low cost, and other advantages [4,5,6,7,8,9,10]. Nowadays, NIR has been widely utilized in the testing of agricultural products [11,12,13,14,15,16,17,18,19], food engineering [20,21], and many other fields. Fan et al. [22] extracted the NIR hyperspectral image of red jujube and built a model based on thermometric methods to identify the types of red jujube in 2017. Zhang et al. [23] employed NIR spectroscopy and partial least squares discriminant analysis (PLSDA) to identify the red jujube varieties in 2017. Luo et al. [24] established an online NIR spectral correction model for the jujube quality of Southern Xinjiang in 2012. Guo, Gu, Liu, & Shang [25] (2016) can identify peach varieties with 100% classification accuracy by least squares support vector machine (LSSVM) and extreme learning machine (ELM) combined with NIR spectroscopy. The genetic algorithm (GA) was utilized to research the NIR spectra of grapes, and the classification accuracy of different grape varieties attained 96.58% [26]. PLSDA combined with local algorithm was employed by Sánchez et al. [27] to classify and recognize strawberry varieties in 2012. Pérez-Marín et al. [28] (2010) employed PLSDA in conjunction with spectral data to accurately classify plum varieties.

Fuzzy recognition is an analytical method which uses fuzzy mathematics theory to solve related problems. Compared with other pattern recognition methods, fuzzy recognition has the advantages of good stability and can accurately describe the diversity of sample information. At present, fuzzy set theory has been used in many fields. Yan et al. [29] combined the maximum boundary criterion (MMC) with fuzzy set theory and proposed a new algorithm-fuzzy maximum boundary criterion. Huang et al. [30] applied fuzzy *k*-nearest neighbor algorithm (FKNN) to face recognition and obtained high accuracy. Xie et al. [31] applied the fuzzy method to spectral extraction, thus providing a new idea and method for two-dimensional optical fiber spectral extraction. Few scholars have applied the fuzzy feature extraction algorithm in the classification of red jujube before. Traditional feature extraction methods lack the description of the diversity of sample class information. Fuzzy pattern recognition is characterized by the complete representation of sample information and good discriminant stability. Traditional LDA always has the problem of small sample size and rank limit, which restrict the extraction of discriminant information, but improved linear discriminant analysis (iLDA) can solve these two problems based on exponential scatter matrixes [32]. Moreover, iLDA can also identify the valid discriminant information in the null space of the within-class matrix Sw, and LDA cannot do this. Fuzzy improved linear discriminant analysis (FiLDA), the combination of fuzzy theory and iLDA, was not only an innovation in fuzzy feature extraction algorithm, but also the better performance than iLDA in dealing with NIR spectra containing noise, so it can improve the classification accuracy of different types of red jujube. At the same time, based on the advantages of iLDA algorithm and exponential fuzzy scatter matrixes, FiLDA can not only overcome the two problems existing in the LDA algorithm, but also solve the problem of sample class information diversity due to the fuzzy theory. FiLDA is an innovative fuzzy feature extraction algorithm which can carry out more accurate feature extraction from NIR spectra containing noise.

LDA is a supervised pattern recognition technology and it is also an effective feature extraction and dimensionality reduction technology [33]. Beverage, liquor, and other fields have been large-scale use of LDA to identify different varieties [34,35,36]. For many applications, the dimensionality of data exceeds the number of data, i.e., the small sample size problem, which may lead to the singularity of the within-class scatter matrix. However, classical LDA requires the within-class scatter matrix to be nonsingular, which is its limitation [37]. Therefore, LDA has been improved in many aspects by researchers. iLDA is feature extraction and dimensionality reduction algorithms that based on LDA, and this can overcome the above problem.

The purpose of this experiment was to combine fuzzy set theory and feature extraction algorithms to establish a classification model for identifying the red jujube varieties. The experimental steps were described as follows: (1) employ a portable NIR spectrometer to collect the spectra of red jujube samples; (2) preprocess the spectral data, and then use feature extraction algorithms to extract features from the data; (3) utilize KNN to build the identification model of red jujube samples, in order to realize the rapid identification of different red jujube varieties.

## 2. Materials and Methods

### 2.1. Sample Preparation

There are five varieties of red jujube samples which come from five production areas (Henan, Shanxi, Xinjiang, Hebei and Gansu) in China. That is, one variety corresponds to one production area. Each variety has 60 samples, so a total of 300 samples were selected. Subsequently, all of the red jujube samples were divided into training and test samples in a certain proportion. The selection of red jujube samples was needed to meet the following requirements: the size (length: 3–5 cm, width: 2–3 cm), weight (10–20 g) and maturity of red jujube which came from the same variety had little difference. Meanwhile, the experimenters ensured that the surface of the red jujube was clean and free from obvious defects.

### 2.2. Spectra Collection

**Figure 1:**
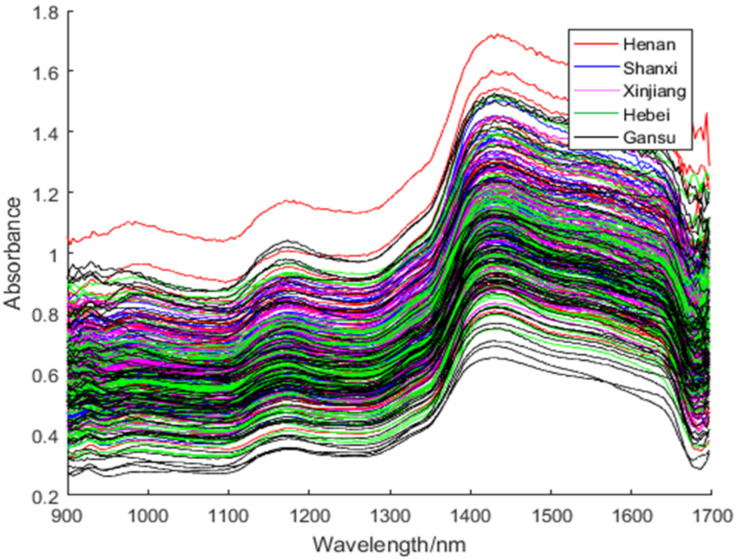
The raw spectra of red jujube samples.

The NIR-M-R2 spectrometer (Shenzhen Pynect Science and Technology Co. Ltd., Shenzhen, China), a portable spectrometer, was employed to collect NIR spectral data of red jujube samples. It has a wavelength range of 900–1700 nm, a signal-to-noise ratio of 6000:1, the InGaAs detector, and a slit size of 1.8 × 0.025 mm. During the whole collection process, the experimental temperature and relative humidity were kept at about 25 °C and 50–60%, respectively. Before collecting the NIR spectral data, the spectrometer must be preheated for one hour. The wavelength range of the collected NIR spectra was 900–1700 nm, and the resolution ratio was 10 nm. The collected NIR spectra of red jujube were the 228-dimensional data. Each red jujube sample was scanned three times by the spectrometer along around the equator, and the final data were the average of the three test results. FiLDA can deal with noisy data better than LDA and iLDA, so we used the whole range of the spectra to show this advantage of FiLDA. The final spectrogram was displayed in Figure 1.

### 2.3. NIR Spectra Preprocessing 

The original spectra were easily influenced by the physical properties of the samples. The data shown in Figure 1 not only had the required sample characteristics but also were mixed with unnecessary information and noise [38]. Therefore, it was necessary to preprocess the spectra to achieve the purpose of enhancing the stability of the model [39]. 

**Figure 2:**
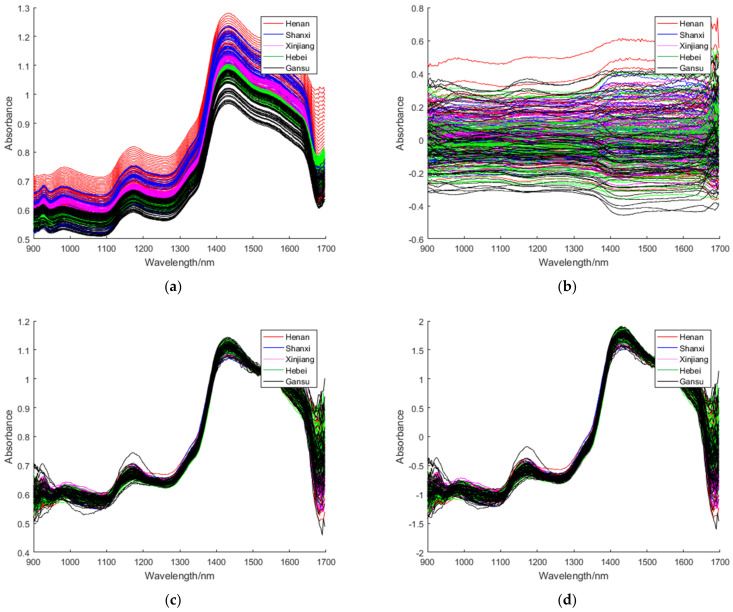

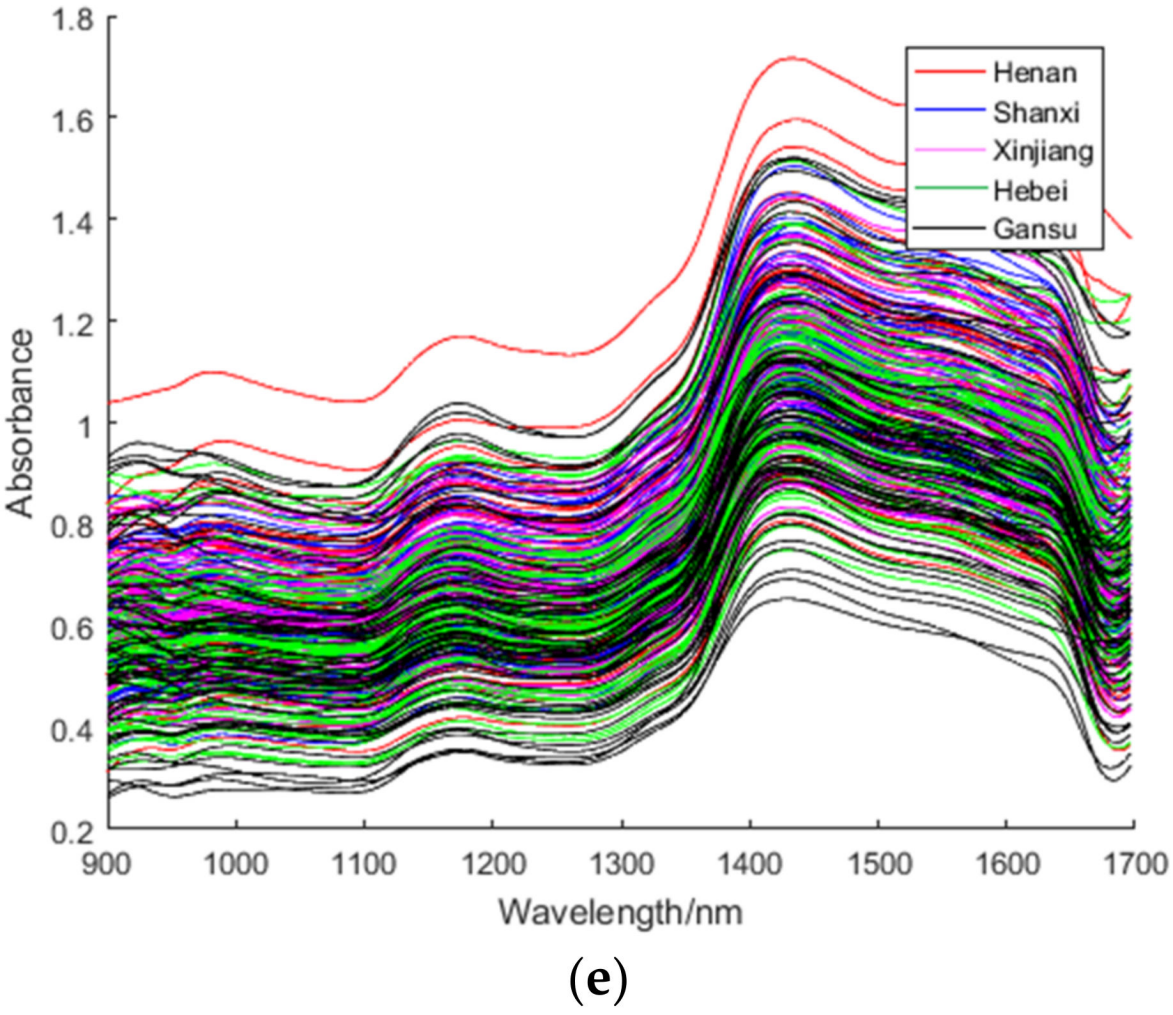
NIR spectra of red jujube samples under different pretreatment methods: (**a**) S-G filter, (**b**) MC, (**c**) MSC, (**d**) SNV, (**e**) S-G smoothing.

In order to get the best experimental results, we employed five pre-processing methods which include MSC, SNV, S-G smoothing, MC and S-G filter [40,41] to preprocess the spectra. For S-G filter, we used Matlab function y = sgolayfilt (x, order, framelen). If x is a matrix, sgolayfilt operates on each column. The polynomial order must be less than the box length framelen, so framelen must be odd. If order = framelen − 1, the filter is not smoothed. In this experiment, the polynomial order was 2 and the box length framelen was 53. Their functions were, respectively, to eliminate scattering phenomenon, reduce the impact of diffuse reflection, decrease random error, delete redundant data and so on. Figure 2 showed the NIR spectra data of red jujube samples after the pre-treatment.

### 2.4. Data Analysis Methods

#### 2.4.1. Principal Component Analysis

The dimensionality of the collected red jujube NIR spectra was 228. These initial NIR spectra of red jujube samples included some redundant information and noise data, which could increase the difficulty of classification and reduce the accuracy of classification. In order to obtain the effective information of NIR spectra, it was necessary to extract multiple eigenvalues for analysis. However, excessive eigenvalues would not only affect the subsequent spectral analysis but also increase the difficulty of the experiment. The purpose of dimensionality reduction is to find characteristic value which can directly mirror the discrepancy of NIR spectra. PCA is a widely used analytical method, which can be employed to reduce dimension and remove redundant information [42,43]. Meanwhile, PCA preserves the characteristic information of NIR spectra by selecting the original eigenvalues [44].

#### 2.4.2. Linear Discriminant Analysis

LDA is a traditional algorithm to reduce the spectral dimension [45]. In the dimensionality reduction process, it uses the prior knowledge and experience of the samples [46]. The ultimate purpose of LDA is to project spectral data from the higher dimensional space to the lower dimensional space, maximize the distance between classes and minimize the distance within classes. 

#### 2.4.3. Improved Linear Discriminant Analysis

iLDA is also an algorithm for feature extraction and it can extract the identification information in the matrix of *S_w_* when the eigenvalues are zero [36].

In this study, iLDA algorithm had two purposes: on the one hand, since the NIR spectra of red jujube was the high-dimensional data, iLDA was employed to deal with spectral data. On the other hand, it could also extract characteristic information from spectral data. Then, the steps of the iLDA are listed as follows (Input: data matrix *D*; Output: transformation matrix *W*):

Step 1. Define the matrices $S_{t}$, $S_{b}$ and $S_{w}$;

Step 2. *B←*${\left(exp\left(S_{w}\right)\right)}^{-1}exp\left(S_{b}\right)$;

Step 3. Eigen decomposition of *B* as $B=UVU^{T}$;

Step 4. $W\leftarrow U_{q}$*,* q=c−1;

In Step 1, three matrices called total scatter matrix *S_t_*, between-class matrix *S_b_*, within-class matrix *S_w_* are shown as follows.

$$S_{t}=\overset{n}{\underset{i=1}{\sum }}\left(d_{i}-\bar{d}\right){\left(d_{i}-\bar{d}\right)}^{T}$$

$$S_{b}=\overset{c}{\underset{j=1}{\sum }}\left(v_{j}-\bar{d}\right){\left(v_{j}-\bar{d}\right)}^{T}$$

$$S_{w}=\overset{c}{\underset{j=1}{\sum }}\;\overset{\;}{\underset{d\in D_{j}}{\sum }}\left(d-v_{j}\right){\left(d_{i}-v_{j}\right)}^{T}$$

Here, $d_{i}$ is the *i*th sample; *c* represents the number of types of experimental samples; *n* is the number of samples; The mean of all the samples is $\bar{d}$; $v_{j}$ denotes the mean value of class j samples in the sample set.

#### 2.4.4. Fuzzy Improved Linear Discriminant Analysis

The steps of the FiLDA are listed as follows (Input: data matrix *D*; Output: transformation matrix *W*):

1. Define the matrices $S_{ft}$, $S_{fb}$ and $S_{fw}$;
2. *B←*${\left(exp\left(S_{fw}\right)\right)}^{-1}exp\left(S_{fb}\right)$;
3. Eigen decomposition of *B* as $B=UVU^{T}$;
4. $W\leftarrow U_{q}$*,*q=c−1;

Three matrices called fuzzy total scatter matrix $S_{ft}$, fuzzy between-class matrix $S_{fb}$ and fuzzy within-class matrix $S_{fw}$ are shown as follows:

$$S_{ft}=\overset{c}{\underset{j=1}{\sum }}\;\overset{n}{\underset{i=1}{\sum }}u_{ij}^{\eta }\left(d_{i}-\bar{d}\right){\left(d_{i}-\bar{d}\right)}^{T}$$

$$S_{fb}=\overset{c}{\underset{j=1}{\sum }}\;\overset{n}{\underset{i=1}{\sum }}u_{ij}^{\eta }\left(v_{j}-\bar{d}\right){\left(v_{j}-\bar{d}\right)}^{T}$$

$$S_{fw}=\overset{c}{\underset{j=1}{\sum }}\;\overset{n}{\underset{i=1}{\sum }}u_{ij}^{\eta }\left(d_{i}-v_{j}\right){\left(d_{i}-v_{j}\right)}^{T}$$

where c is the number of sample categories and n is the number of training sample data. $u_{ij}$ is the fuzzy membership value of the *i*th data point. η is the weight index. FiLDA algorithm is a combination of fuzzy membership function and iLDA algorithm; it cannot only describe the diversity of sample information but also solve the small sample size problem of LDA. 

#### 2.4.5. K Nearest Neighbor

KNN is a supervised pattern recognition algorithm whose basic principle is that the same kind of experimental samples are close to each other, and the different kinds of experimental samples are far away from each other [47]. 

We employed PCA + LDA, PCA + iLDA, and PCA + FiLDA to realize feature extraction on NIR spectra and then we used the KNN algorithm to establish a classification model of red jujube varieties. The classification accuracy of the model would be affected by the number of samples and the internal parameter K in the course of trying to establish the test model.

### 2.5. Software

In this article, all of the algorithms were performed using Matlab 2014a (The MathWorks, Natick, MA, USA).

## 3. Results and Discussion

### 3.1. Spectral Analysis

In this study, the wavelength scope of the collected NIR spectra of red jujube was 900–1700 nm. The NIR spectra contained a lot of characteristic functional group information as shown in Figure 1. There are 2 distinct peaks, which are 1180 nm and 1430 nm, in the NIR spectra of red jujube samples. After 1350 nm, the absorbance of all of the red jujube samples dramatically changes, which is due to the absorption of O-H and water [48]. From Figure 1, we can also find that the absorbance of the red jujube samples reaches the peak of the whole spectrum at 1430 nm. The first part is connected with the first and second frequency multiplications of C-H group stretching vibration. These absorptions reflect protein-like substances. The peak at 1430 nm may be related to the first and second order frequency doubling of the O-H group in the water [49]. Since red jujube samples with five different varieties have different functional group information, the NIR spectra were able to accurately express all of the samples.

### 3.2. Spectral Preprocessing

Figure 2 showed the NIR spectra of red jujube samples under different pre-processing methods. These pre-processing methods were employed in this article: S-G smoothing, S-G filter, MC, MSC and SNV. Compared with other spectra, the spectra (b) pre-processed by MC had no obvious peaks and troughs, while the red jujube spectra pre-processed by the other methods all showed obvious peaks and troughs. We tried five preprocessing methods to deal with NIR spectra and found S-G filter with the best effect, so we choose S-G filter to preprocess the spectra in this paper. After spectral pre-processing, we applied PCA + LDA, PCA + iLDA and PCA + FiLDA to implement feature extraction on NIR spectra. The classification accuracy of jujube variety under PCA + LDA, PCA + iLDA and PCA + FiLDA were introduced below.

### 3.3. Classification with PCA + LDA

**Figure 3:**
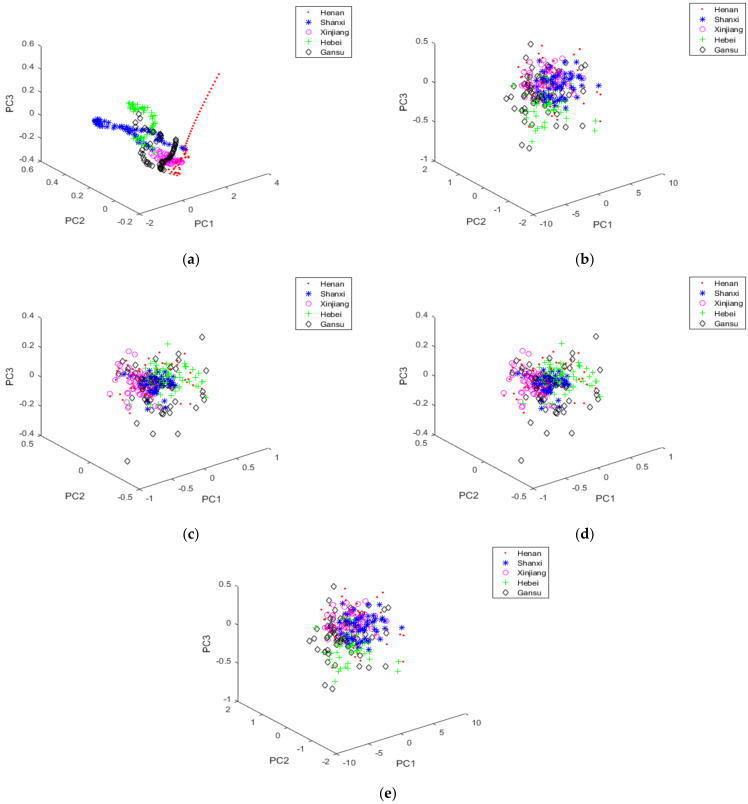
PCA scores plot of vectors with PC1, PC2 and PC3 under different pretreatment methods: (**a**) S-G filter, (**b**) MC, (**c**) MSC, (**d**) SNV, (**e**) S-G smoothing.

The data cannot be used directly after pre-processing because the spectral data contained a lot of repetitive information. This phenomenon was unfavourable for the classification of red jujube varieties. Therefore, in order to obtain the principal components of the spectrum of red jujube samples and remove the redundant information, the spectral dimension must be reduced first [11]. In this experiment, the cumulative contribution of the first 7 principal components was more than 99.98%, and then the NIR spectral data was projected into the first seven principal components, which could improve the classification accuracy of the experiment. Moreover, the eigenvalues were as follows: λ_1_ = 133.189, λ_2_ = 7.711, λ_3_ = 7.258, λ_4_ = 0.425, λ_5_ = 0.117, λ_6_ = 0.062, λ_7_ = 0.029. Since the first 3 principal components (PC1, PC2, and PC3) accounted for 99.6% of the total square deviation, they not only preserved the characteristic information of the NIR spectrum data but also eliminated the redundant information. Therefore, the three-dimensional feature space of NIR spectral data of red jujube was established. Figure 3 displayed the PCA scores plot of vectors with PC1, PC2, and PC3. Since the experiment used different pre-treatment methods, the spectra of red jujube after PCA treatment were different. It could be seen from the Figure 3 that the clustering positions of each kind of red jujube sample were different, so it was proved that the feature extraction algorithm could be used to classify and identify red jujube from different origins. Among them, the classification effect of Figure 3a was the best, and the classification effect of Figure 3b was the worst. Then the accumulative eigenvalue of PC1 accounted for 89.9% for those of the first 3 principal components (PC1-PC3). Additionally, it was easy to find that the red jujube samples still could not be well recognized by PCA. Therefore, in order to get a better classification effect, it was necessary to adopt more feature extraction methods to obtain the identification information from red jujube samples. In this experiment, PCA + LDA is a two-stage algorithm. That is to say, PCA is employed to reduce the dimension of spectral data, and then LDA is applied to extract the characteristic information of spectral data. Therefore, PCA was employed to reduce the dimensionality of the red jujube NIR spectral data to 7 latent variables. Then, LDA was responsible for extracting discriminant information and the test samples were mapped to these discriminant vectors of LDA. 

**Figure 4:**
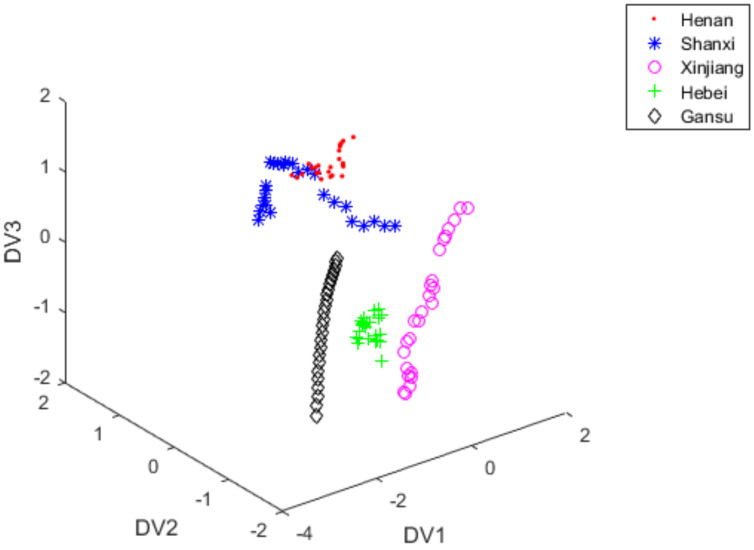
LDA scores plot of vectors with DV1, DV2 and DV3 under S-G filter +PCA + LDA.

LDA scores plot of vectors with DV1, DV2, and DV3 were shown in Figure 4. In Figure 4, samples in 2 varieties of red jujubes (Henan and Shanxi) overlapped each other, but most of the experimental samples of red jujube could be easily distinguished.

### 3.4. Classification with iLDA

**Figure 5:**
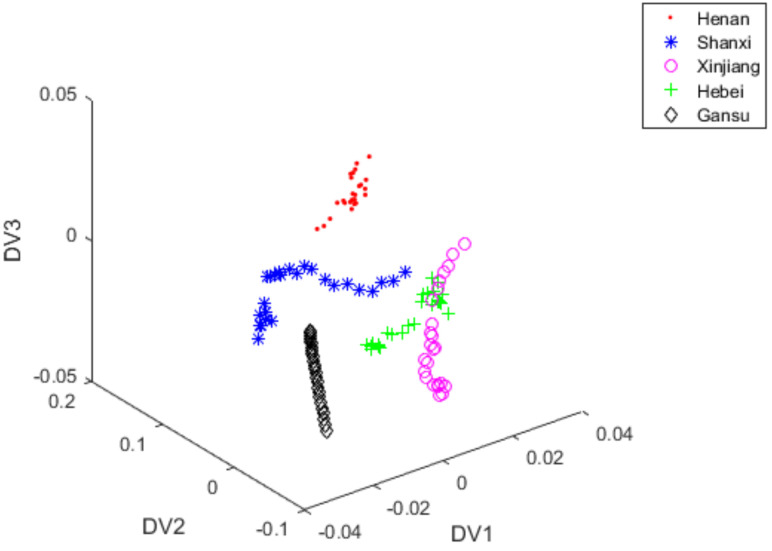
iLDA scores plot of vectors with DV1, DV2 and DV3 under S-G filter +PCA + iLDA.

iLDA extracted discriminant information from the 7-dimensional spectral data. A total of 300 red jujube samples were divided into the training set (each variety of red jujube has 35 training samples, totally 175) and the test set (each variety of red jujube has 25 test samples, totally 125). After the training set was processed by iLDA to produce 3 optimal discriminant vectors (DV1, DV2 and DV3), the 7-dimensional spectral data of 125 test samples were projected to DV1, DV2 and DV3. Figure 5 showed the scores plot of three optimal discriminant vectors. As shown in Figure 5, test samples of the NIR spectral data had good distribution. However, there were 13 samples from Hebei misclassified as those from Xinjiang and there were 10 samples from Shanxi misclassified as those from Henan. There were 3 samples from Xinjiang misclassified as those from Shanxi, and there was also 1 sample from Gansu misclassified as that from Hebei. Therefore, its classification accuracy was only 77.6%. 

### 3.5. Classification with FiLDA

In this section, FiLDA was applied to extract feature information of the NIR spectral data after PCA dimension reduction. All of the parameters were as follows: the fuzzy weight parameter η=4, the number of sample categories c=5. The initial cluster centers of FiLDA were:

$$V^{\left(0\right)}=\left[\begin{array}{c} v_{1}^{\left(0\right)}\\ v_{2}^{\left(0\right)}\\ v_{3}^{\left(0\right)}\\ v_{4}^{\left(0\right)}\\ v_{5}^{\left(0\right)} \end{array}\right]=\left[\begin{array}{c} 0.9550\;\\ 0.3765\;\\ -0.1315\;\\ -0.2947\;\\ -0.9564\; \end{array}\begin{array}{c} -0.1284\;\\ 0.2290\;\\ -0.0897\;\\ 0.0378\;\\ -0.1167\; \end{array}\begin{array}{c} 0.1512\;\\ -0.0789\;\\ -0.0984\;\\ 0.0864\;\\ 0.0497\; \end{array}\begin{array}{c} -0.0214\;\\ -0.0086\;\\ 0.0295\;\\ 0.0084\;\\ -0.0397\; \end{array}\begin{array}{c} -0.0184\;\\ 0.0002\;\\ -0.0005\\ -0.0038\\ 0.0112\; \end{array}\begin{array}{c} 0.0084\;\\ 0.0074\;\\ -0.0121\;\\ -0.0206\;\\ 0.0120\; \end{array}\begin{array}{c} 0.0049\\ -0.0066\\ 0.0164\\ -0.0033\\ 0.0033 \end{array}\right]$$

**Figure 6:**
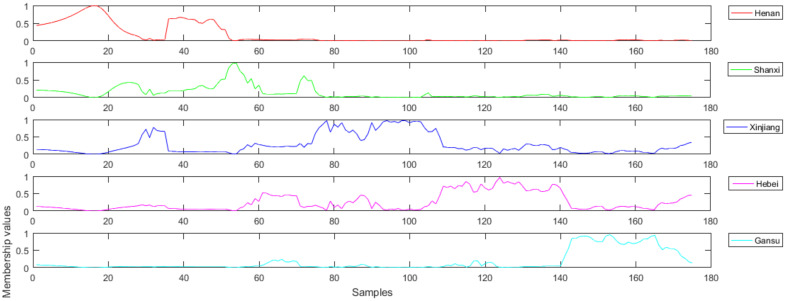
Initial fuzzy membership values.

The initial fuzzy membership values of FiLDA were displayed in Figure 6. The abscissa represented sample set and the ordinate signified fuzzy membership values. There were five different varieties in this experiment, so there were five different little figures. Each little figure represented red jujube from one origin, and they represented Henan, Shanxi, Xinjiang, Hebei, and Gansu, respectively. When the value of the ordinate exceeds 0.5, it means that the test sample belongs to the red jujube of a certain origin. When the fuzzy membership value of the *i*th sample $u_{ij}$ was the biggest in the *j*th class, we could confirm the *i*th sample belonged to the *j*th class.

**Figure 7:**
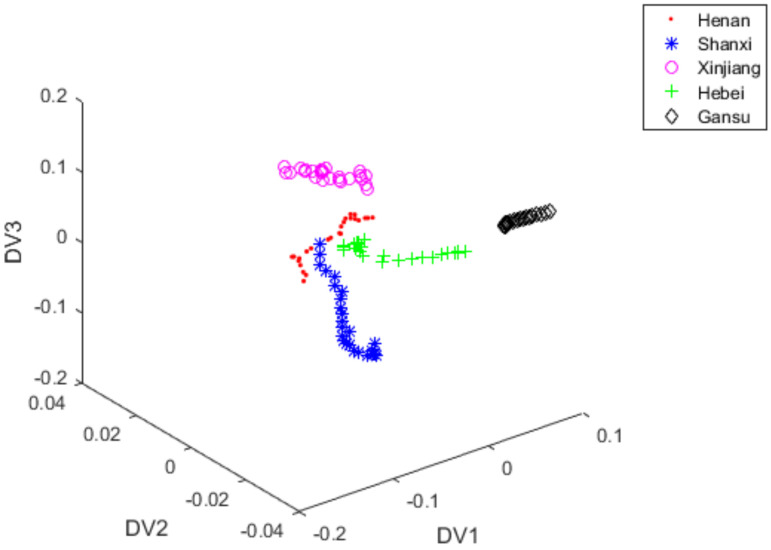
FiLDA scores plot of vectors with DV1, DV2 and DV3 under S-G filter + PCA + FiLDA.

Figure 4, Figure 5 and Figure 7 used the first seven PCs to develop discriminant analysis model and S-G filter was applied as pre-processing method. Figure 7 displayed the three-dimensional scoring diagram when the feature extraction algorithm of FiLDA was used to extract the identification information from the test set of red jujube samples. A total of 5 different kinds of red jujube samples could be clearly identified by using FiLDA with the classification accuracy 94.4%. In view of classification results, the data distribution of Figure 7 was obviously better than that in Figure 5. This further demonstrated the effectiveness of FiLDA in extracting the identification information from NIR spectra of red jujube. 

### 3.6. Classification Results of KNN

Table 1 displayed the recognition accuracies of red jujube varieties from different origins by using several pre-processing methods and feature extraction algorithms. At the same time, other conditions remain unchanged (especially the number of training samples n_training is 175 and the number of testing samples n_test is 125). 

The pre-processing method and feature extraction algorithm were S-G filter and LDA, respectively, and the classification accuracy of the KNN was 75.2%. There were 14 samples from Shanxi misclassified as those from Henan and there were also 4 samples from Xinjiang misclassified as those from Shanxi. There was also 11 sample from Hebei misclassified as that from Xinjiang, and there were also 2 samples from Gansu misclassified as those from Hebei. The pre-processing method and feature extraction algorithm were S-G filter and FiLDA, respectively, and the classification accuracy of the KNN reached 94.4%. There were 2 samples from Hebei misclassified as those from Shanxi, and there were also 2 samples from Gansu misclassified as those from Hebei. There was also about 1 sample from Shanxi misclassified as that from Henan and there was also 1 sample from Xinjiang misclassified as that from Shanxi. It can prove that FiLDA can classify red jujube varieties and has a good classification effect. At the same time, it was apparent that the classification accuracies of LDA were generally not as good as those of iLDA and FiLDA when using the same pre-processing methods.

### 3.7. Discussion

The NIR spectral data were collected by the NIR-M-R2 spectrometer, and then spectral data were processed by S-G filter, PCA, LDA, iLDA and FiLDA. Then, KNN was applied to classify the test samples. We evidently discovered that the classification accuracies of red jujube varieties were different when different feature extraction algorithms were used in the experiments in Table 1. The classification accuracies reached less than 90% when the PCA + LDA/iLDA were employed as feature extraction algorithms. In contrast, they could reach more than 90% when the PCA + FiLDA was applied as feature extraction algorithm. As was shown in Table 1, it could be found that the classification accuracy was the highest when both FiLDA and the S-G filter preprocessing method were utilized in this classification system for processing NIR spectra of red jujube samples.

The number of training samples and test samples was changed, but other experimental conditions were consistent. Table 2 displayed the classification accuracies of red jujube varieties by several feature extraction methods and different number of training data and test data. In Table 2, n_training indicates the number of training samples, and n_ test represents the number of test samples. It was easy to find that the classification accuracies changed with the above 2 parameters. From Table 2, we could clearly see that PCA + FiLDA can better classify different kinds of red jujube samples compared with PCA + LDA/iLDA. When the parameters of n_training and n_test were 175 and 125, respectively, the classification accuracy of PCA + FiLDA also reached the highest with 94.4%.

## 4. Conclusions

To classify red jujube varieties quickly, nondestructively, and effectively, FiLDA algorithm coupled with NIR spectroscopy was proposed in this study. FiLDA algorithm is the derivation of fuzzy set theory and iLDA. FiLDA is a new fuzzy feature extraction algorithm that combines the fuzzy algorithm with the iLDA, and it is applied in the identification of red jujube varieties. The NIR spectral data were collected for 300 red jujube samples of 5 types by using the NIR-M-R2 spectrometer. NIR spectra were processed by S-G filter, PCA, LDA, iLDA and FiLDA, respectively. Finally, KNN was employed as a classifier to recognize the red jujube varieties. FiLDA was able to identify red jujube samples accurately and had the highest classification accuracies than other feature extraction algorithms. In addition, NIR spectroscopy has been widely used in the field of food inspection, and in the food supply chain. The experimental results proved that FiLDA algorithm coupled with NIR spectroscopy could play an important role in the classification of red jujube varieties.

## Figures and Tables

**Table 1:** Classification accuracies by several preprocessing methods and feature extraction algorithms.

	SNV	MSC	MC	S-G Smoothing	S-G Filter
PCA + LDA	47.2%	44.0%	44.6%	45.6%	75.2%
PCA + iLDA	50.1%	44.0%	47.2%	58.4%	77.6%
PCA + FiLDA	52.5%	68.5%	62.5%	75.0%	94.4%

**Table 2:** Classification accuracies with different number of training data and test data.

n_training	n_test	PCA + LDA	PCA + iLDA	PCA + FiLDA
150	150	77.3%	79.3%	92.0%
175	125	75.2%	77.6%	94.4%
200	100	75.0%	76.0%	90.0%

## Data Availability

Data sharing is not applicable to this article.
